# Portomesenteric Thrombosis Secondary to Acute Cholecystitis: A Case Report

**DOI:** 10.1155/2018/9409081

**Published:** 2018-08-08

**Authors:** Haseeb Ahmad Chaudhary, Ibrahim Yusuf Abubeker, Kamran Mushtaq, Khaldun Obeidat, Anand Kartha

**Affiliations:** Department of Medicine, Hamad Medical Corporation, Doha, Qatar

## Abstract

Portomesenteric venous thrombosis (PMVT) is an uncommon clinical problem. Common risk factors include intra-abdominal infections, abdominal surgeries, malignancy, cirrhosis, and inherited thrombophilia. Early recognition and treatment of PMVT are important to avoid serious complications like mesenteric ischemia and infarction. Acute cholecystitis is a clinical condition encountered daily but rarely may be complicated by development of portomesenteric venous thrombosis. Only few cases have been reported of superior mesenteric vein thrombosis secondary to cholecystitis. We report a case of a forty-one-year-old male patient who developed partial portal and superior mesenteric vein thrombosis after mild acute cholecystitis for which surgery had been deferred. Patient had no other identifiable risk factors for thrombosis. Patient was successfully treated with 6 months of anticoagulation with warfarin and complete recanalization of portomesenteric veins was achieved at the end of treatment.

## 1. Introduction

The prevalence of portal vein thrombosis is about 1% in the general population [1]. Wide variety of causes of portal and superior mesenteric vein thrombosis has been reported before [2]. The three main categorical groups are malignant thrombosis, cirrhotic PVT, and nonmalignant, noncirrhotic PVT [3]. The most common risk factors for PVT are cirrhosis, hepatobiliary malignancies, and pancreatitis [4]. Acquired thrombotic risk factors, such as latent myeloproliferative disorders and prothrombotic genetic defects, have also been identified as major risk factors for PVT [5]. Early recognition of PMVT is important as delayed diagnosis can lead to life threatening complication like mesenteric ischemia and infarction.

## 2. Case Presentation

We report a case of a forty-one-year-old male patient who presented to our emergency department with chief complaints of abdominal pain and was found to have right upper quadrant tenderness. There was no significant past medical, psychosocial, and family history. Ultrasound of abdomen showed distended gallbladder wall, with wall thickness measuring 7 mm along with pericholecystic fluid suggestive of acute cholecystitis. In addition, a 7 mm calculus was also noted in the cystic duct. Common bile duct diameter was 4 mm and portal vein trunk diameter was 10 mm. A hypodense lesion 11 by 15 mm was also seen in the left lobe of liver suggesting hemangioma. He was diagnosed with mild acute calculous cholecystitis and was discharged on oral antibiotics. He was advised for interval cholecystectomy in 4 weeks.

Sixteen days later, he presented again to the emergency with periumbilical, postprandial abdominal pain. It was associated with nausea and vomiting but no fever, jaundice, or change in bowel habits. On examination, his vital signs were normal, and abdomen was soft with minimal right hypochondrial tenderness, there was no hepatosplenomegaly, and bowel sounds were normal. There was no melena on digital rectal exam.

Laboratory investigation revealed WBC: 6500 x 109/L, Hb:159 gm/l, and PLT:247000 x10^9^/L. Coagulation studies including prothrombin time, partial thromboplastin time, and INR were normal, and urea, creatinine, and electrolytes were all within normal range. Liver function tests revealed ALT: 29 IU/L, AST: 17 IU/L, ALP:117 IU/L, total bilirubin: 6 umol/l, protein:76 gm/l, and albumin: 41gm/l and CRP was very elevated at 1476 nmol/L (range: 0.76-28.5 nmol/l).

**Figure 1:**
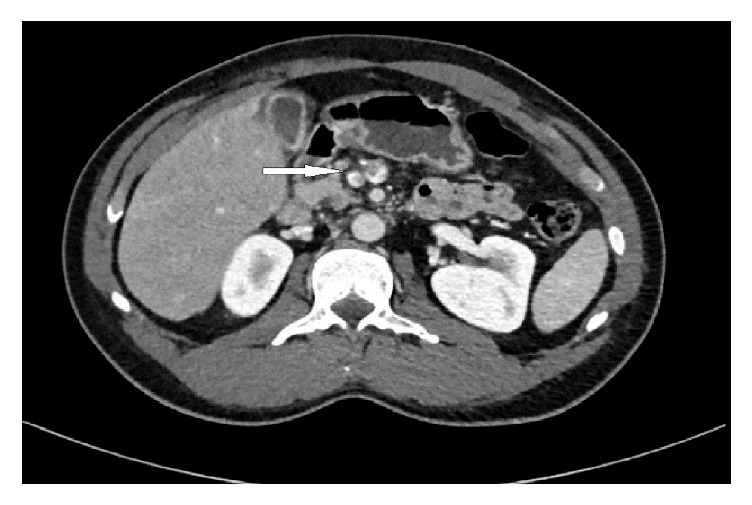
Contrast-enhanced CT scan showing pericholecystic fluid and partial filling defect in the superior mesenteric vein (white arrow).

**Figure 2:**
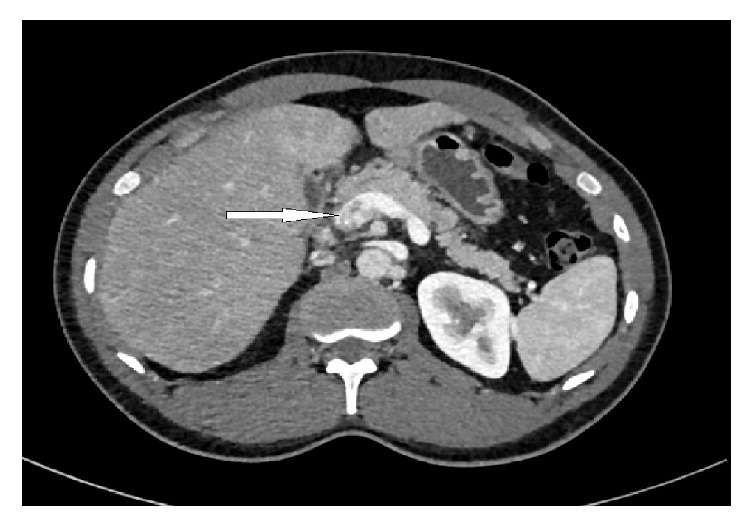
Contrast-enhanced CT scan abdomen showing partial filling defect in the portal vein (white arrow).

A contrast-enhanced CT scan of the abdomen was performed to rule out any complications as the changing nature of pain was not explained by cholecystitis alone. Apart from confirming the pericholecystic fluid and distended gall bladder, it also showed filling defects in several branches of the superior mesenteric vein and portal vein confluence with partial obliteration of the lumen, suggesting venous thrombosis, and part of the distal small bowel loops demonstrated apparent wall thickening with hyperenhancement and mesenteric congestion (Figures 1 and 2).

Doppler ultrasound study of hepatobiliary system also confirmed the presence of partial thrombosis. Portomesenteric thrombosis is an unusual site for thrombosis so work-up was done to rule out other causes. Antithrombin III activity was 101.2% (normal range 71-116 %), homocysteine: 8 umol/L (range: 5-15 umol/L), and ANA, anticardiolipin IgG, and IgM antibodies were negative. Genetic testing for prothrombin gene mutation 20210 and factor V Leiden mutation was also negative. Flow cytometric analysis of peripheral blood for paroxysmal nocturnal hemoglobinuria was negative. JAK2 mutation was not detected. Alpha-fetoprotein level was normal. MRI abdomen was performed to assess the nature of hypoechoic lesion in the liver seen on the initial ultrasound. MRI abdomen confirmed that the hypoechoic lesion in right lobe of liver was hemangioma and possibility of a primary liver tumour was ruled out.

**Figure 3:**
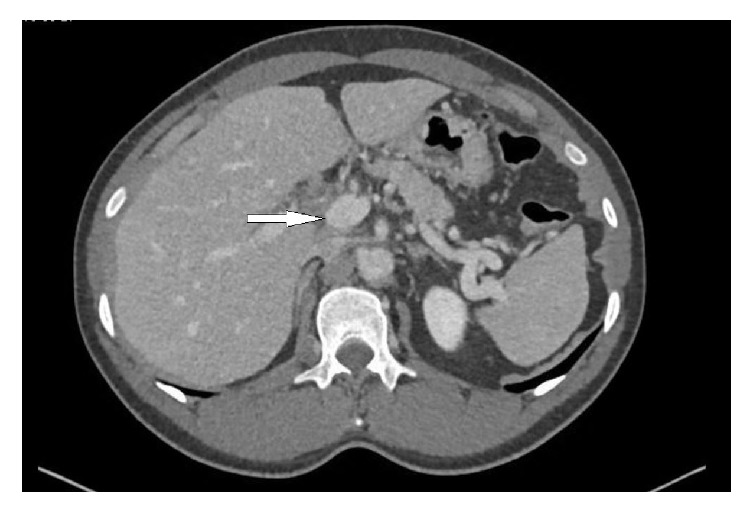
Contrast-enhanced CT abdomen after 6 months of anticoagulation showing complete recanalization of previously seen portomesenteric thrombosis.

The patient was started on therapeutic anticoagulation with enoxaparin at 1 mg/kg subcutaneous BID dose and IV Ceftriaxone 2 grams per day along with bowel rest. After 24 hrs the patient was started on warfarin and enoxaparin was continued for 5 days for overlap until his INR was in therapeutic range (2.0-3.0). Patient was discharged after 6 days of hospitalization and appointment with surgical team for cholecystectomy was given. His stay in the hospital was uneventful so a repeat CT scan was not done to look for bowel ischemia. On follow-up at 6 months patient was doing well clinically and completed the warfarin course. Repeat CT abdomen with contrast showed complete recanalization of portal and superior mesenteric veins (Figure 3) and patient is waiting for cholecystectomy.

## 3. Discussion

Portomesenteric venous thrombosis (PMVT) includes thrombosis involving portal vein and superior mesenteric and/or inferior mesenteric vein. Acute portal or superior mesenteric vein thrombosis often presents with abdominal pain, whereas chronic disease manifests either as an incidental finding on CT or with features of portal hypertension. Contrast-enhanced CT scan of abdomen diagnoses more than 90% of cases and is considered gold standard as it can also diagnose potential complications like mesenteric ischemia and infarction [6]. Our patient's initial presentation with mild acute cholecystitis and later presentation with central abdominal pain led to the decision of contrast-enhanced CT imaging that revealed partial thrombosis of portal and superior mesenteric veins. In contrast to the reported cases before [7], our patient did not have septic cholecystitis with absence of fever, leukocytosis, and near-normal liver function tests on presentation indicating the cholecystitis was of mild nature with raised CRP and US features suggestive of such a diagnosis [8].

Local inflammatory causes of PMVT cholecystitis, cholangitis, hepatitis, appendicitis, diverticulitis, and pancreatitis have all been reported in the past [3]. One-third of these patients have more than one risk factor for thrombosis [9]. Given the vague presentation, high degree of clinical suspicion is required to diagnose patients with PMVT [10]. PMVT remains undiagnosed in several cases and detected incidentally during examination for other reasons [6]. PMVT have also been reported after several intra-abdominal surgeries in which case surgery itself acts as a risk factor for thrombosis [11]. Studies have suggested the incidence of portal vein thrombosis to be higher with laparoscopic surgery than open surgery [12].

PMVT is a rare complication of acute cholecystitis as previously reported; our case is unique in a way that our patient had mild form of cholecystitis and did not undergo surgery and still had portal vein thrombosis which was more extensive and partially involved the superior mesenteric vein as well. The early recognition of this complication of a mild cholecystitis with appropriate medical management can improve outcomes [13] and shorten hospital stay [14]. Prompt recognition is also critically important to avoid life threatening complications like mesenteric ischemia and infarction [15].

Work-up for inherited and acquired thrombophilia conditions was negative, with normal antithrombin III level and homocysteine levels. Advanced imaging with CT and MRI did not reveal any local tumour that could have contributed to the thrombus formation and propagation. The indication to perform MRI Liver was to characterize the hypodense lesion picked up by ultrasonogram in the left lobe of liver, which proved to be a hemangioma. Our patient did not have any evidence of haemolysis but flow cytometry for paroxysmal nocturnal hemoglobinuria was performed to rule out that possibility, JAK2 mutation was also not detected which ruled out polycythaemia vera and essential thrombocytosis [16].

We believe the initial triggering factor for portal vein thrombosis in our case could be the intense inflammatory response caused by the stone in the cystic duct, which sits in close proximity to the draining cystic veins in Calot's triangle [17]. Cystic veins eventually drain into the right portal vein branch through which the thrombosis can propagate if left untreated, although the ultrasound Doppler did not reveal dilatation in the cystic vein in this case.

Nevertheless, with bowel rest, IV hydration, antibiotics, and anticoagulation patient's condition dramatically improved and he was discharged pain-free from the hospital with follow-up. There is newer data emerging on use of direct acting oral anticoagulants and novel oral anticoagulants (NOACs) in PMVT [18, 19]. However, we restricted anticoagulation to warfarin due to the affordability issues of our patient.

## 4. Conclusion

Portomesenteric vein thrombosis is a rare complication of acute cholecystitis. Early recognition of this complication of cholecystitis with appropriate medical management can improve outcomes and shorten hospital stay and prevent life threatening complications like mesenteric ischemia. PMVT can be successfully treated with oral anticoagulation with warfarin resulting in favourable outcome.
